# Ectropion Repair Techniques and the Role of Adjunctive Superotemporal Skin Transposition for Tarsal Ectropion

**DOI:** 10.3390/jcm14030827

**Published:** 2025-01-27

**Authors:** Brendan K. Tao, Thanansayan Dhivagaran, Fahad R. Butt, Michael Balas, Ahsen Hussain, Navdeep Nijhawan, Georges Nassrallah, Edsel Ing

**Affiliations:** 1 Faculty of Medicine, The University of British Columbia, Vancouver, BC V6T 1Z3, Canada; brendan.tao2016@gmail.com; 2 Schulich School of Medicine & Dentistry, University of Western Ontario, London, ON N6A 5C1, Canada; tdhivagaran2027@meds.uwo.ca (T.D.); fbutt2027@meds.uwo.ca (F.R.B.); 3 Department of Ophthalmology and Vision Sciences, University of Toronto, Toronto, ON M5S 2L9, Canada; 1michaelbalas@gmail.com (M.B.); gnassra1@jhmi.edu (G.N.); 4 Department of Ophthalmology, Dalhousie University, Halifax, NS B3H 2Y9, Canada; ahsen@dal.ca; 5 Department of Ophthalmology and Visual Sciences, University of Alberta, Edmonton, AB T5H 3V9, Canada

**Keywords:** oculoplastic surgery, superotemporal skin transposition, tarsal ectropion, recurrent ectropion, eyelid

## Abstract

**Background:** Ectropion is a common eyelid problem and is defined as eversion of the eyelid margin and typically involves the lower eyelid. The main acquired causes of ectropion include involutional, cicatricial, paralytic, and mechanical. A severe manifestation of ectropion is tarsal ectropion, where much of the tarsal conjunctiva is visible, often with keratinization of the conjunctiva. causes. Common techniques for ectropion repair include horizontal tightening of the lid with lateral tarsal strip or Bick procedure, lateral tarsorraphy, inverting sutures and the sub-orbicularis oculi fat lift. However, all surgical techniques are prone to ectropion recurrence. We review the techniques for ectropion repair and describe a novel adjunctive technique called the superotemporal skin transposition (STS), which is well suited for patients with recurrent or tarsal ectropion. **Methods:** The STS is combined with a lateral tarsal strip or Bick procedure. For the STS, all of the anterior lamellae of the lateral lower lid is retained. The posterior lamellae is sutured to the lateral orbital tubercle. A triangular bed of skin is excised superotemporally, and the lower lid anterior lamellae is transposed and secured with multiple sutures. The STS can be combined with inverting sutures, or skin graft for cicatricial cases. **Results:** We used the STS with Bick procedure and optional inverting sutures on 23 patients, 4 of whom required bilateral ectropion repair. At 1–6 month followup all patients achieved satisfactory outcomes with a well-positioned eyelid and improved symptoms. The STS had more lateral cutaneous scarring than with a Bick procedure alone, but patients did not find this objectionable. No reoperations were required. **Conclusion:** The STS is a straightforward and useful adjunct for patients with severe, recurrent or tarsal ectropion. Further studies are needed to determine the long-term efficacy of this technique.

## 1. Introduction

Ectropion is eversion of the eyelid margin and predominantly occurs within the lower lid. Ectropion can result in corneal exposure, tearing conjunctival erythema, and eye irritation. The most commonly acquired causes of ectropion are involutional, cicatricial, paralytic and mechanical. Tarsal ectropion refers to marked eversion of the lower lid, which may be associated with keratinization of the palpebral conjunctiva. We review the indications, benefits and limitations of the established surgical technique for ectropion repair. These procedures include the lateral tarsal strip (LTS) procedure and Bick procedure to correct horizontal lid laxity, tarsorrhaphy or inverting sutures for disinsertion of the lower lid retractors, and skin grafts for cicatricial ectropion. The indications for and steps of superotemporal skin transposition (STS) as an adjunct for the treatment of severe or tarsal ectropion are described. Advanced techniques such as the sub-orbicularis oculi Fat (SOOF) lift for cicatricial ectropion or midface descent, and the vertical-to-horizontal rotational myocutaneous flap for cicatricial ectropion are briefly reviewed.

## 2. Surgical Techniques for Ectropion

Table 1 presents a series of tarsal ectropion repair procedures, including their indications, specific techniques, and rates of success in conjunction with their follow-up duration, which vary across studies [1,2,3,4,5,6]. In this section, we discuss common surgical techniques for ectropion.

Horizontal lid-tightening techniques for ectropion include the LTS, Bick procedure, Kuhnt–Szymanowski and occasionally medial canthoplasty [7]. A video illustration of LTS is documented elsewhere [8]. The procedure begins with a lateral canthal incision to provide access, followed by an inferior cantholysis to release the lower canthal tendon. If medial or punctal ectropion is present, a medial spindle procedure can be performed as an adjunct [9]. In this step, a diamond-shaped section of the eyelid retractors and conjunctival tissue is excised just below the punctum, and then closed with an inverting suture that is externalized through the skin inferiorly. The tarsal strip is measured to ensure the appropriate length, after which the epithelium is debrided to prepare the tarsal strip. Next, the anterior and posterior lamellae are divided, followed by separating the tarsal strip from the lower eyelid retractors and conjunctiva. The anterior lamella is excised so that lash follicles are removed from the tarsal strip. The tarsal strip is then reattached to the periosteum at the lateral orbital tubercle with subsequent reshaping of the lateral canthus such that its proper angle is recovered. The use of a specialized needle, such as a m4-0 Vicryl S-2 semicircular needle, is advantageous for securing a precise bite of the inner rim of the lateral orbital wall, which aids in achieving optimal lower lid positioning. This technique helps achieve the lid globe apposition for optimal procedural outcomes. A 5-0 non-absorbable prolene suture may be advisable for recurrent ectropion, to decrease recurrence. In patients with tarsal ectropion, reverse Quickert sutures can be used as an adjunct [10]. This approach uses double-ended 5-0 chromic sutures, passed from below the tarsus and exiting near the lid–cheek junction, to plicate the retractors and enhance lower eyelid positioning. These sutures are typically placed in pairs, with the tension adjusted based on the degree of lid margin ectropion, often with a preference for slight overcorrection to ensure stability in both entropion and ectropion cases. Finally, the skin is sutured closed [7].

Medial canthoplasty is another horizontal tightening procedure that may be used in cases of ectropion with medial canthal tendon laxity. This surgical technique begins with an incision made inferior to the lacrimal canaliculus, which continues medially to the medial canthus and runs superior to the tendon. Next, the surgeon passes a mattress suture with 4-0 Prolene into the tarsus at the nasal aspect and subsequently passes it through the periosteum inferior to the insertion of the superficial arm of the medial canthal tendon [11,12,13].

Bick’s shortening is a horizontal tightening technique similar to the lateral tarsal strip. This procedure involves the full-thickness excision of a triangular segment of eyelid tissue near the lateral canthus of the affected eyelid. The extent of tissue resection is carefully tailored to the severity of the eyelid laxity. Following the excision, the edges of the resected eyelid tissue are precisely realigned to generate the necessary horizontal tension. Sutures are then placed through the tarsus to secure the newly adjusted eyelid position, with meticulous closure of the skin layers and optional closure of the conjunctiva and muscular layers as needed to optimize healing [14]. Compared to traditional surgical techniques such as LTS, Bick’s shortening method can be simpler to perform and has demonstrated superior anatomical and functional outcomes in ectropion management [14]. However, the technique has been criticized for potentially causing rounding and medial displacement of the canthal angle [14].

The Kuhnt–Szymanowski procedure involves shortening the horizontal lid with a full-thickness pentagonal wedge resection combined with a lateral lid incision approximately 3 mm from the lid margin. The authors perform the Bick and LTS procedures in preference to the Kuhnt–Szymanowski procedure, as the former are quicker and do not cause a lid margin notch.

Canthal suspension techniques include lateral canthal sling and tarsorrhaphy [12]. The lateral canthal sling procedure is employed to address cases of paralytic ectropion [15]. This condition can be further worsened by periocular aging or increased eyelid laxity, in which the lateral canthal tendon loosens, resulting in eyelid sagging and ectropion. The lateral canthal sling aims to provide support and restore tension in the lateral canthal tendon. In this technique, a small horizontal incision is made near the lateral canthus along the natural eyelid crease, exposing the lateral canthal tendon. The surrounding soft tissue is then dissected away to mobilize adjacent structures, including the tarsal plate and orbicularis muscle. An autologous or synthetic sling material is then threaded or looped around the canthal tendon and anchored to a stable structure around the orbital rim, often the periosteum of the lateral orbital rim for increased support. Finally, the sling is adjusted to achieve appropriate tension, realigning the lower eyelid in its proper anatomical position [15,16].

A small lateral tarsorrhaphy can help suspend the lid in patients with severe cases of paralytic ectropion and increased eyelid laxity, particularly when prior surgical management has failed or when eyelid exposure poses a risk to globe integrity [2]. Tarsorrhaphy involves partially suturing the upper and lower eyelid margins near the central or lateral canthus, with sutures that may be permanent or temporary depending on case severity. By decreasing the inferior scleral show, tarsorrhaphy reduces corneal desiccation and irritation. Tarsorraphy may serve as either temporary protection until more definitive reconstructive procedures are feasible or as a permanent solution when other management options are contraindicated or inadequate [2].

In some cases, ectropion may require skin grafts or Mustardé flaps. Particularly, in cases of cicatricial ectropion, which is often due to anterior lamellar tissue scarring, it may be necessary to remove the scarred area and support the anterior lamella deficiency with a skin graft. The upper eyelid, supraclavicular region, preauricular or postauricular areas and volar forearm are common hairless donor sites that may be harvested for the skin graft. Full-thickness skin grafts (FTSGs) include epidermis and dermis layers, whereas split-thickness skin grafts (STSGs) involve the epidermis and only a portion of the dermis. FTSGs are most commonly used to surgically manage cicatricial ectropion and provide a greater match to eyelid skin in terms of color and texture compared to STSGs. FTSGs demonstrate up to a 100% graft survival rate across several studies and are associated with lower rates of secondary contraction compared to STSGs; however, there is a theoretical risk of failure which may be influenced by individual patient characteristics [17,18,19]. STSGs are used less frequently for the repair of ectropion and are more likely to contract compared to FTSGs. Given the enhanced cosmetic results and reduced risk of secondary contraction, FTSGs are typically preferred over STSGs in cases of cicatricial ectropion repair [17,18,20]. Skin grafting begins with releasing the scarred tissue and subsequent harvesting of the graft from a donor site. The graft is shaped appropriately to the dimensions of the defect, secured into position with sutures and traction sutures. A pressure dressing is applied for 5–7 days postoperatively.

Marked ectropion can also be managed through a sub-orbicularis oculi fat (SOOF) lift, particularly in cases of paralytic ectropion [21]. It is indicated in cases with age-related descent of the midface structures and severe eyelid laxity [21]. This procedure provides structural support to the lower eyelid by lifting the orbicularis muscle pad. It begins with an incision on the interior aspect of the lower eyelid, about 1 mm below the caudal margin of the tarsal and through the orbicularis oculi muscle to expose the SOOF. The SOOF is released from the surrounding structures and lifted into a position higher along the orbital rim, where it is secured with sutures to the periosteum [21]. This procedure can be augmented with a lateral canthal tightening procedure to further reinforce the eyelid position.

Finally, a technique for treating cicatricial or severe ectropion, described by Chang et al., is the vertical-to-horizontal rotational myocutaneous flap (VHRMF) based on the Tsai procedure. “A vertical myocutaneous flap is created from the anterior lamella of the vertical pedicle in the lateral third of the lower eyelid. Following a horizontal relaxing incision from the base of the flap, a vertical myocutaneous flap is created and rotated to horizontal” [22]. The advantages of this technique include reduced donor site morbidity and correction of horizontal lid laxity [22]. For patients with cicatricial ectropion, the cicatrix still has to be released, whereby midface lifts can be performed and anterior lamella graft may still be required. The VHRMF technique may surpass the comprehensive ophthalmologist’s technical repertoire and, in some cases, may be accompanied by a visible vertical scar [22].

## 3. Superotemporal Skin Transposition

Superotemporal skin transposition is a versatile surgical technique within the purview of the comprehensive ophthalmologist and general plastic surgeon that may be used as an adjunct for the repair of tarsal and recurrent ectropion from involutional or paralytic causes. When performed with skin grafts, it can also be applied to cicatricial ectropion. This technique conserves the anterior lamella skin that is normally discarded during traditional LTS or Bick ectropion repairs and provides a superotemporal vector to the eyelid repair. A triangular bed of skin over the lateral orbital rim superior to the lateral canthus is excised. A skin flap from the inferolateral lid is transferred superotemporally. The skin flap allows for better lid globe apposition and structural support. Although the STS may cause a vertical scar in the lateral orbit area, it is not seen frontally, as may occur with the VHRMF.

We illustrate the application of the superotemporal skin-transposition technique for managing paralytic ectropion, underscoring its advantages over conventional methods in providing enhanced support and esthetic results.

## 4. Illustrative Case and Technique of STS

**Figure 1:**
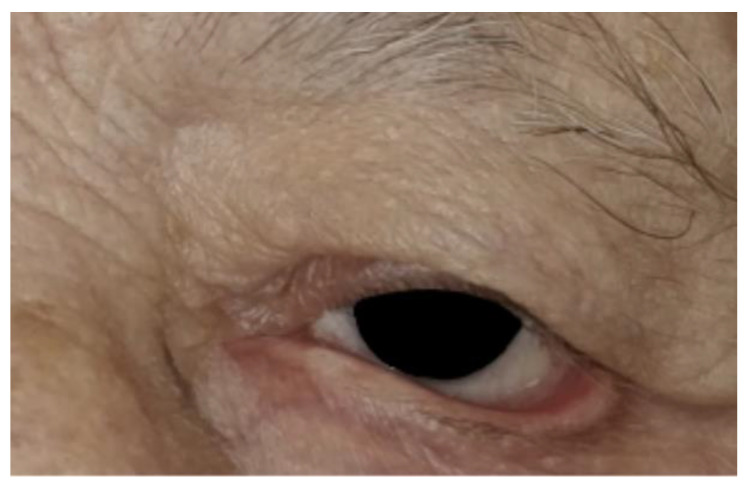
Preoperative appearance of the patient with ectropion in the left eye.

An elderly male patient presented with symptomatic left paralytic ectropion (Figure 1). The patient reported issues with exposure-related symptoms, including irritation, tearing, and visual disturbances.

**Figure 2:**
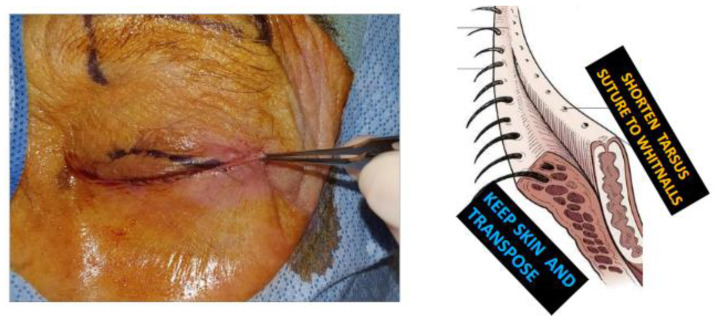
Initial incision and eyelid split—the eyelid was split with preservation of the skin, except at the lash margin, to expose the posterior lamella for later stabilization.

Following lateral canthotomy and cantholyis, the lateral lid was split into an anterior and posterior lamella. Unlike the LTS/Bick procedures, all the lateral lid skin was retained except at the lateral lid margin. The posterior lamella was shortened to create optimal tension and sutured to Whitnall’s ligament (Figure 2).

**Figure 3:**
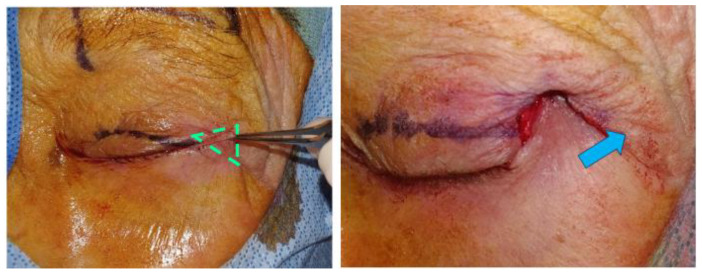
Preparation of superotemporal skin bed—an excision bed underneath the skin flap (green triangle) was created lateral and superior to the canthus to accommodate the transposed skin flap. The blue arrow illustrates the lateral and superior vector of STS that augments wound healing and stability.

**Figure 4:**
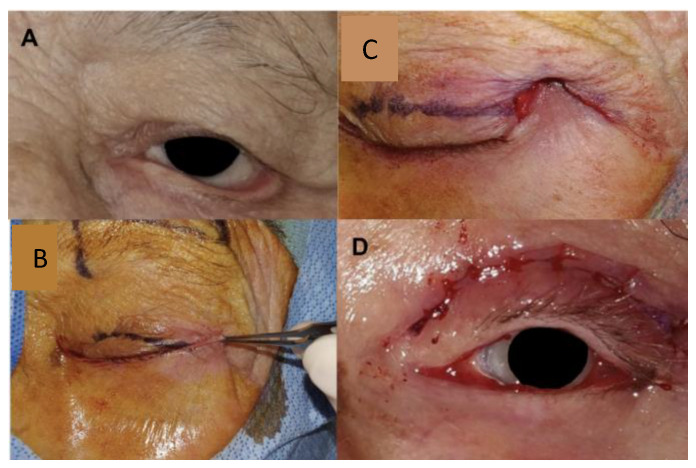
Summary of the steps in the superotemporal transposition procedure and the final appearance—(**A**) Pre-operative appearance in a patient with involutional and paralytic tarsal ectropion (**B**) The retained skin was used as a template for the amount of superotemporal skin to excise. Not visualized underneath, the posterior lamella is sutured to the lateral orbital tubercle. (**C**) The anterior lamella was transposed and suspended in the prepared superotemporal bed and secured with interrupted sutures. (**D**) The lower lid margin is well repaired. A gold weight and direct brow lift were also performed.

Next, the skin over the lateral orbital rim superior to the lateral canthus was marked using the preserved eyelid skin flap as a template. (Figure 3) The superolateral skin was excised, and the preserved anterior lamella skin was transposed into the superotemporal bed. The transposed skin flap was positioned to fall into the natural contour, aiming for functional and esthetic restoration. Multiple interrupted sutures were placed to secure the transposed flap into the superolateral bed (Figure 4). In this case, which involved paralytic ectropion, a brow lift and upper-lid gold weight were also inserted.

The patient’s postoperative care included standard guidelines for ectropion repair, and monitoring for infection and healing.

The superotemporal skin transposition augmentation has benefits and drawbacks compared to the traditional surgical management options for ectropion (Table 2) [1,2,4,5]. Compared to conventional techniques such as LTS or Bick’s shortening, adjunctive superotemporal skin transposition provides a stronger, more stable repair by reducing tension on the posterior lamella, allowing stronger adhesion. However, it requires a slightly longer procedure time with an increased risk of bleeding and ecchymoses, and the potential for mild lateral scarring or rhytid formation. When compared to the augmented LTS technique alone, the superotemporal transposition maintains better lid globe apposition. Compared to tarsorrhaphy, the superotemporal transposition offers improved cosmesis of the lid fissure and preserves peripheral vision, though it is more challenging to perform. Finally, when compared to the SOOF lift, the superotemporal technique is easier to perform and suitable for patients on anticoagulants. However, the SOOF lift does not have temporal scarring and serves as a valuable management option for cases involving significant midface descent or age-related laxity.

**Figure 5:**
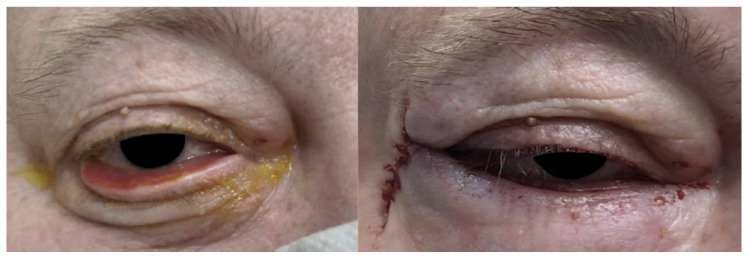
Pre- and immediate postoperative photos of a patient with involutional tarsal ectropion. The surgical technique was a Bick procedure and skin transposition without inverting sutures.

**Figure 6:**
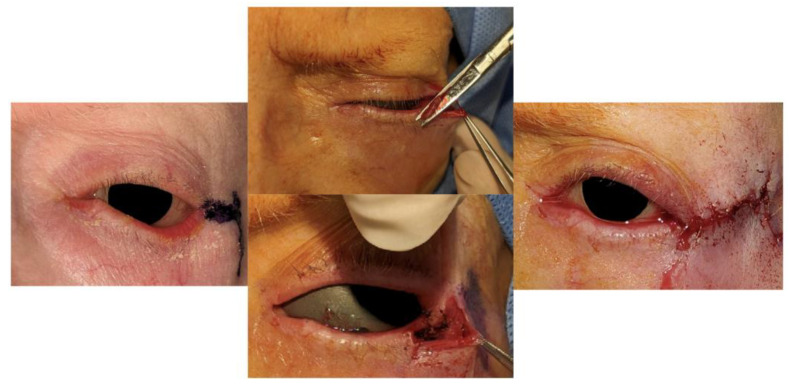
This patient with tarsal ectropion and skin cicatrix had residual punctal ectropion. In retrospect, inverting sutures and a skin graft could have been added.

These points demonstrate the efficacy of the superotemporal skin transposition technique in managing tarsal ectropion. By providing stable lid positioning, improved functional outcomes, and a favorable aesthetic result, this approach is a viable adjunct to conventional surgical options. The senior author (EI) has used the STS with a modified Bick’s procedure and optional inverting sutures on 23 patients (see Figure 4, Figure 5 and Figure 6). Four patients required bilateral ectropion repair. In the short-term follow-up period (1–6 months), all patients achieved outcomes that were rated as satisfactory or better on the grading scale used by Berry-Brincat, with a well-positioned eyelid with improved symptoms [1]. No reoperations were required, though some patients experienced mild residual eversion. Mild lower eyelid ectropion, unlike entropion, does not always necessitate surgical repair in patients with normal upper lid blink (i.e., non-paralytic cause), intact corneal sensation and a functional Bell’s phenomenon. Given these criteria, unstable or otherwise unsuitable patients may safely defer or avoid ectropion repair, provided that adequate eye lubrication and eye monitoring.

## 5. Conclusions

Tarsal ectropion is not uncommon, and all oculofacial surgeons should have a repertoire of surgical repair techniques based on the underlying etiology and severity of the case. This review highlights the effectiveness of these techniques and introduces an adjunctive superotemporal skin-transposition technique. This procedure is performed with horizontal lid tightening with the option of inverting sutures and offers enhanced lid stability compared to traditional procedures. The skin transposition requires about five additional minutes of operating time, and presents minor risks of scarring and ecchymoses. In our pilot case series, this method has demonstrated patient satisfaction and favorable outcomes in early follow-ups. However, as this series was conducted within the past 3.5 years, longer-term outcomes over several more years await investigation. In our experience, although patients were instructed to follow up if postoperative issues arose, all patients to date declined further follow-up given their satisfaction with the surgical outcome. Further studies are needed to establish the long-term efficacy of this procedure.

## Figures and Tables

**Table 1:** Summary of commonly used techniques for managing tarsal ectropion by indication and success rates with corresponding follow-up durations.

Series	Technique	Indication	Success Rate—Proportion (%)	Follow-Up Duration in Months—Mean (Range)
Kwon et al., 2015 [4]	LTS + 5 mm Lat. Tarsorraphy	Paralytic	22/22 (100%; 3 patients did not report satisfactory cosmesis)	16.5 (12–73)
Wang et al., 2022 [6]	Inverting sutures + mod Bick	Involutional	31/34 (91%)	4.5 (3–11)
Berry-Brincat et al., 2013 [1]	Inverting sutures ± LTS	Involutional	18/20 (90%)	3.6 (2–15)
Neavyn et al., 2012 [5]	Inverting sutures ± LTS	Involutional, Recurrent	6/7 (86%)	2 (1–4)
Chung et al., 2007 [3]	Midface lift + LTS	Cicatricial, Involutional, Paralytic	17/22 (78%)	12 (2–38)
Chang et al., 2006 [2]	Augmented Tarsal Strip	Paralytic	13/14 (93%)	21 (6–30)

**Table 2:** Technique comparison: superotemporal skin transposition vs. other techniques for ectropion correction.

Technique	Indications	Advantages	Disadvantages	Success Rate
Superotemporal Skin Transposition	Cicatricial, recurrent ectropion, and cases requiring robust lid stabilization	Provides a strong and stable repair by reducing tension on the posterior lamella Preserves peripheral vision with cosmetically favorable results Safer for patients on anticoagulants	Longer operative time Risk of lateral scarring and ecchymosis Technically challenging	Initial follow-up (1–6 months): 100% satisfactory outcomes
Conventional LTS/Bick	Primarily involutional ectropion, moderate horizontal laxity cases	Effective for moderate horizontal laxity correction High success rate in involutional ectropion Simplifies procedure with significant anatomical outcomes	Limited correction for severe cicatricial cases Can affect peripheral vision	90–100% in several studies; reference [4]
Augmented LTS	Paralytic and severe involutional ectropion	Provides enhanced stability and support for the eyelid, especially in cases with severe laxity Can better preserve anatomical alignment and minimize postoperative complications like lid retraction	More technically complex than conventional LTS Longer operative and recovery time	Reported success rate: 93%; reference [2]
Tarsorrhaphy	Severe paralytic ectropion with risk of corneal exposure	Provides strong eyelid support in high-risk cases, reducing the risk of corneal exposure Limits eyelid opening, helping to protect the eye surface from desiccation	Significantly reduces peripheral vision May negatively impact cosmetic appearance, often noticeable due to eyelid fusion	85% in high-risk cases; reference [5]
SOOF lift	Cases with significant midface descent or age-related laxity	Enhances midface elevation and provides additional support to lower eyelid Improves esthetic appearance in patients with midface laxity	Not suitable for patients on anticoagulants due to bleeding risk Temporal scarring can occur, potentially affecting esthetics	Success rate varies; suitable as adjunct; reference [1]

## Data Availability

No data were created during the production of this work.
